# *Clostridium septicum* myonecrosis following gardening: A case report

**DOI:** 10.1016/j.ijscr.2023.108000

**Published:** 2023-03-20

**Authors:** Salik Hamid, Ashok Gadré, Liselott Fornander, Johanna Sjöwall, Måns Muhrbeck

**Affiliations:** a Department of Surgery, Vrinnevi Hospital, Norrköping, Sweden; b Department of Biomedical and Clinical Sciences, Linköping University, Linköping, Sweden; c Department of Anesthesiology and Intensive Care, Vrinnevi Hospital, Norrköping, Sweden; d Department of Infectious Diseases, Vrinnevi Hospital, Norrköping, Sweden

**Keywords:** Myonecrosis, Gas gangrene, Soft-tissue infection, *Clostridium septicum*, Surgery, Case report

## Abstract

**Introduction and importance:**

Clostridial myonecrosis (CM), or gas gangrene, is a rare necrotizing muscle infection caused most often by *Clostridium perfringens* or *C. septicum*. Inoculation can occur either traumatically or spontaneously. CM has a high mortality rate if not treated promptly.

**Case presentation:**

A 64-year-old male presented to the emergency department (ED) with sudden onset left flank pain and fever. Repeated CT scans demonstrated progressive edema around the left iliopsoas muscle with gas formation and bleeding. The patient received intravenous fluids, meropenem, and clindamycin. Emergency laparotomy was performed on suspicion of necrotizing fasciitis and revealed a necrotic left iliopsoas muscle which was partially excised. Blood cultures were positive at 12 h with growth of *C. septicum*. Prolonged stay in the intensive care unit, and six additional surgical interventions to the abdomen, left thigh, and flank were needed. The patient was discharged after four months to a nursing home.

**Clinical discussion:**

*C. septicum* CM more often occurs spontaneously and is associated with colorectal malignancy. However, for our patient, CT colonography and proctoscopy did not reveal any pathology. Therefore, we believe the CM resulted from an injury the patient sustained while working in his backyard, either a cut from barbed wire on his arm or from soil contaminating his psoriatic lesions. Successful outcomes for patients with CM require a high index of suspicion, timely treatment with antibiotics, and repeated surgical debridements.

**Conclusion:**

This case report describes the presentation and management of a presumably injury-related CM caused by *C. septicum*.

## Highlights

• Infectious myonecrosis following an injury may be caused by *C. septicum*.
• Spontaneous clostridial myonecrosis is often associated with colorectal cancer.
• Prompt diagnosis and management are vital for a successful outcome.
• Repeated surgical revisions are essential to achieve source control.

## 1 Introduction

Clostridial myonecrosis (CM), or gas gangrene, is a necrotizing muscle infection caused by bacteria from the anaerobic Clostridium family. Clostridioides inhabit the soil and are part of the normal flora in the human gastrointestinal tract. The most common species causing CM are *Clostridium perfringens* and *septicum* [1]. Both are opportunistic pathogens, causing infections through hematogenous dissemination of resilient endospores to less perfused tissues [2]. *C. perfringens* is associated with CM following traumatic injuries and *C. septicum* with spontaneously occurring CM. Furthermore, spontaneous CM is associated with malignancy and in particular colorectal malignancy. *C. septicum* survives longer in aerobic environments than *C. perfringens*, as it does not require the same strict anaerobic conditions [3].

Historically CM has been a feared complication of war-related injuries, with an incidence of around 5 % during WWI [4]. In civilian health care CM is rare, with an incidence of approx. 1000 cases/year in the United States [5]. Of these, 50 % are associated with traumatic injuries, 35 % occur postoperatively, and 10 % have a spontaneous etiology [6]. The mortality rate is near 100 % if left untreated [7].

This case report describes the history, workup, management, and discharge status of a patient with CM caused by *C. septicum*, presumably caused by a minor injury. The patient was treated at a secondary-care level hospital in Sweden, with a catchment area of 180,000 inhabitants.

This case report was written in compliance with the SCARE 2020 Guideline [8].

## 2 Presentation of case

A 64-year-old male patient with a history of alcohol and tobacco dependency, cured hepatitis C, liver cirrhosis (Child-Pugh score A), right-sided hip replacement, chronic obstructive pulmonary disease (GOLD stage 3B), and psoriasis presented to the emergency department (ED) with sudden onset of left flank pain and fever for the past 8 h. The patient had normal bowel and bladder function and denied any trauma. He had been working in his backyard the day prior.

Findings on initial examination were a temperature of 39.4 °C, an oxygen saturation of 94 %, heart rate (HR) of 120 bpm, and a blood pressure (BP) of 143/75 mm Hg. No abdominal tenderness or guarding was noted; provocation for flank pain was inconclusive. Apart from his non-infected psoriatic lesions, there were no signs of penetrating trauma.

**Fig. 1:**
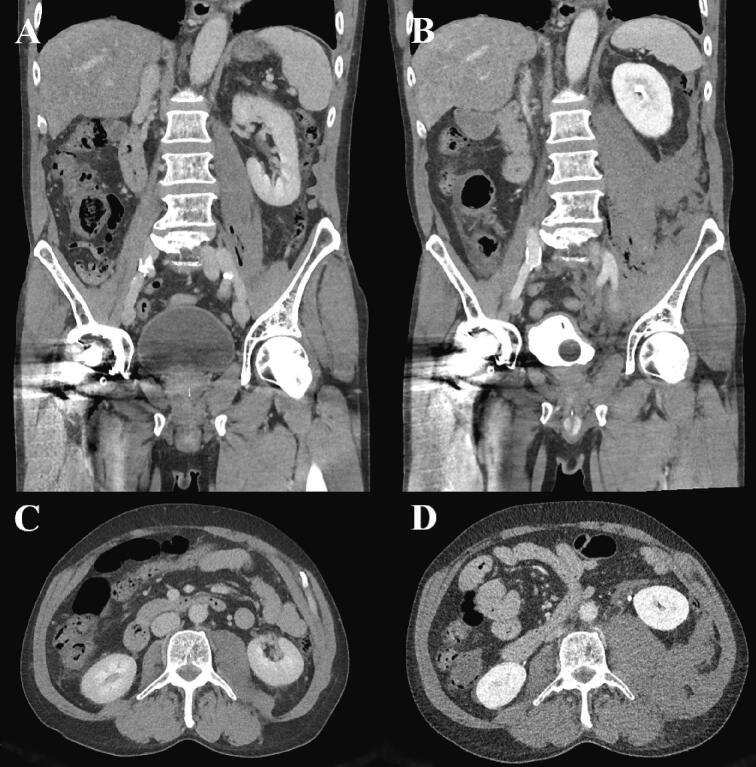
Abdominal CT on day 1. (A) Coronal and (C) axial planes from CT on admission display edema around the left iliopsoas muscle and minor gas formation within the muscle compartment. (B) Coronal and (D) axial planes from CT 2.5 h after admission display progression of the edema and gas formation. The left kidney is displaced anteriorly due to the collection of fluid, attenuating as blood, around the left iliopsoas muscle.

Blood analysis demonstrated C-reactive protein level of 18 mg/L (<10 mg/L), venous lactate 3.8 mmol/L (0.6–2.4 mmol/L), but no leukocytosis. Based on the patient's clinical features tentative diagnosis was intra-abdominal sepsis. Blood cultures were taken, and treatment with intravenous ringer-acetate, meropenem, and clindamycin was initiated. A computer tomography (CT) scan revealed edema around the left iliopsoas muscle with minor local gas formation (Fig. 1).

After evaluation by the on-call surgeon and discussions with the senior radiologist and surgical colleagues, a second CT was done 2.5 h after the first one. Progression of the edema and gas formation was seen. Moreover, fluid attenuating as blood was now present around the left iliopsoas muscle (Fig. 1). The patient had also deteriorated with a HR of 140 bpm, BP of 90/70 mm Hg, and a venous lactate ranging from 3.8 to 4.8 mmol/L. Due to suspicion for necrotizing fasciitis, the patient was taken to the operating theatre (OT) for emergency laparotomy.

**Fig. 2:**
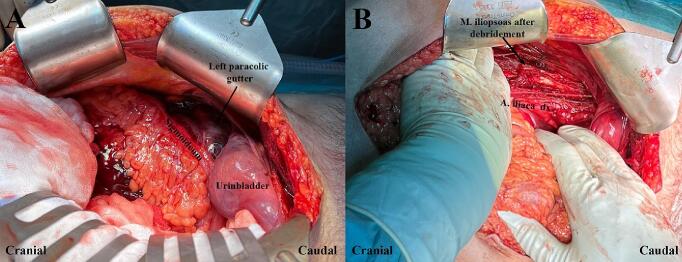
Findings on emergency laparotomy on day 1. (A) An edema of the urine bladder's peritoneal sheet and a retroperitoneal hematoma in the left paracolic gutter were found. (B) Left iliopsoas muscle compartment after left medial visceral rotation, evacuation of hematoma, and debridement of the left psoas muscle.

Anesthesia was induced with ketamine and the patient was orally intubated. Shifting to sevoflurane resulted in a dramatic decrease in blood pressure and anesthesia was therefore maintained with ketamine. A ureteral catheter was inserted intraoperatively to facilitate identification of the left ureter. In the abdominal cavity, edema of the urine bladder's peritoneal sheet and a retroperitoneal hematoma in the left paracolic gutter were found. Left medial visceral rotation was done to evacuate the hematoma and expose the iliopsoas muscle (Fig. 2). Gas bubbles were seen in the swollen and discolored muscle. Muscle samples were taken for bacterial cultures, and a group A streptococcus rapid antigen detection test was performed with negative results. Approx. 50 % of the psoas muscle was necrotic and excised. Passive drains were placed in the space of the left iliopsoas muscle and the pouch of Douglas. The abdomen was temporarily closed with vacuum-assisted wound closure and mesh-mediated fascial traction (VAWCM).

**Fig. 3:**
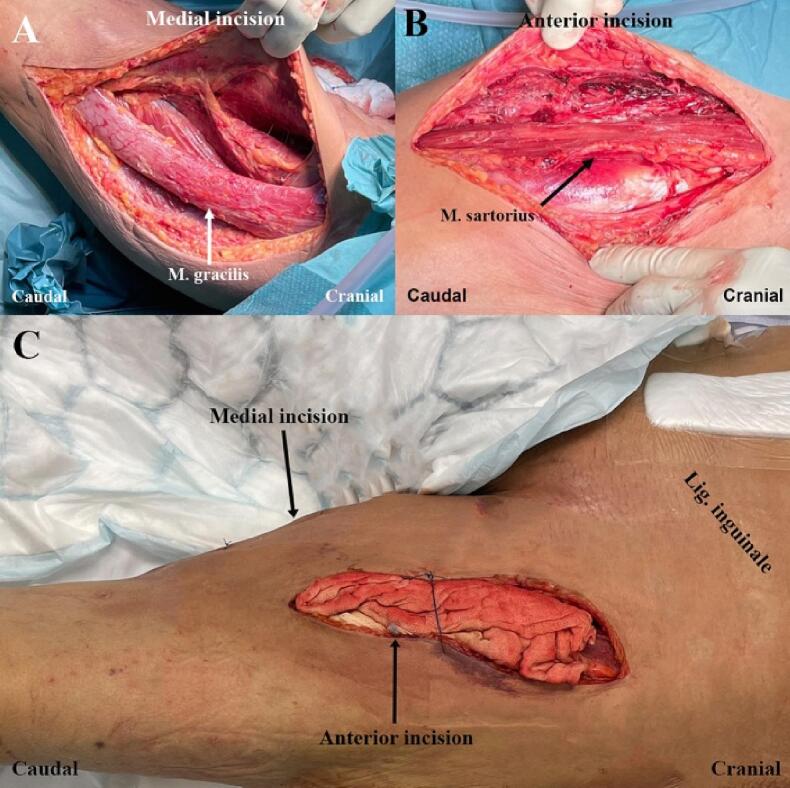
Debridement of the left thigh on day 4. (A) Medial wound after excision of partly necrotic muscles. (B) Anterior wound after excision of superficially necrotic muscles. (C) Dressing of anterior wound with gauze saturated with 30 % hydrogen peroxide.

Postoperatively, the patient was kept on a ventilator and taken to the intensive care unit (ICU). A second laparotomy was performed 18 h after the first. No further debridement of the left psoas muscle was necessary. The abdomen was again closed with VAWCM. The left thigh was incised anteriorly due to newly developed discoloration. Necrotic portions of the sartorius and iliopsoas muscles were excised (Fig. 3). The wound was packed with saline-soaked gauze.

**Fig. 4:**
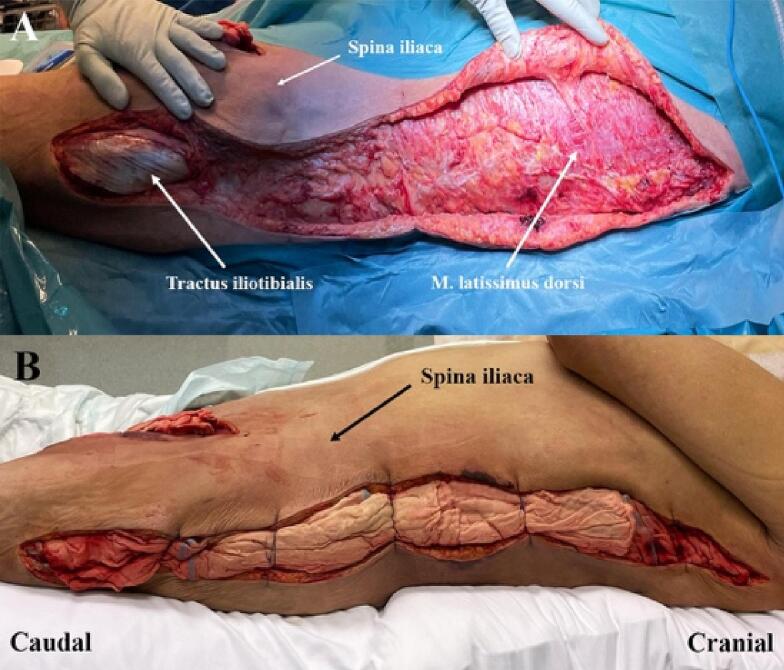
Debridement from the left thigh to the scapula on day 4. (A) Wound after excision of superficially necrotic muscles. (B) Dressing of lateral incision with gauze saturated with 30 % hydrogen peroxide.

On hospital day 2, the blood cultures exhibited growth of *C. septicum*, and metronidazole was added to the antibiotic treatment. At this time, the discoloration of the thigh had progressed. The patient was taken to the OT for a third time. Incisions were made to the medial and lateral aspects of the thigh. The lateral incision was extended to the scapula. Subcutaneous edema was seen, but only superficial part of the muscles was necrotic and thus excised. The wounds were packed with gauze soaked in 30 % hydrogen peroxide. Skin edges were approximated with suture to hold the gauzes in place and facilitate future wound closure (Fig. 4). A third laparotomy was also performed, and the remaining iliopsoas muscle was found to be viable. The abdominal incision was therefore closed. The center for hyperbaric oxygen therapy (HBOT) at Karolinska University Hospital was contacted to inquire if HBOT would benefit the patient. The decision was made not to transfer the patient as the risks associated with the transport were considered greater than the potential benefit of HBOT.

**Fig. 5:**
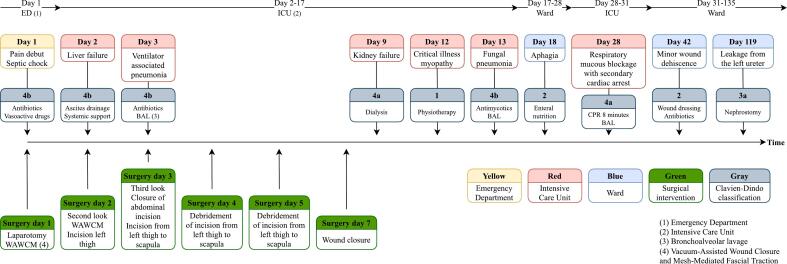
Timeline of level of care, surgical interventions, and complications.

The patient underwent additional minor debridements of the left thigh and flank on hospital days 4 and 5, and the wounds were closed on day 7. The patient was extubated on hospital day 14 and transferred to the Infectious Diseases ward on day 16. While in the ICU and on the ward, the patient suffered several complications (Fig. 5). Most notable was a mucous blockage of the lung with subsequent cardiac arrest (day 28) and intraabdominal leakage from the left ureter (day 119). The latter was most likely caused by residual histolytic effects of CM as creatinine measured in ascites a week after the last abdominal procedure was within normal range. The patient was discharged to a nursing home on day 135 for rehabilitation. Treatment with antibiotics and analgesics had been completed prior to discharge. At discharge, the patient had a slightly lower body mass index than on admission (21.7 and 22.6 kg/m^2^, respectively). He exhibited weak hip flexion on the left-side but was able to walk approx. 20 m with a wheeled walker before needing to rest. Outpatient dressing changes for a minor wound dehiscence and investigation for potential reconstruction of the left ureter were scheduled.

## 3 Discussion

The literature states that spontaneous CM is more often caused by *C. septicum* than *C. perfringens*, and is associated with colorectal malignancy [7], [9]. The hypothesis being that the malignancy causes a mucosal breach allowing bacteria to enter the blood stream [9]. This association was not seen in our patient, as a CT colonography and proctoscopy did not identify any colorectal pathology. Therefore, we believe that the CM resulted from an injury that the patient sustained while working in his backyard. He may have suffered a minuscule cut on his arm while moving barbed wire or soil may have come into contact with his psoriatic lesions.

The onset of CM is swift with intense muscle pain, edema or bullae that contain fluid or blood. Palpable emphysema often occurs at a later stage [2], [10]. Survival requires prompt initiation of treatment. Therefore, a high index of suspicion for CM in patients with fever and severe muscle pain is warranted. Cultures should be obtained before, but must not delay, antibiotic treatment covering pathogens associated with necrotizing fasciitis. Piperacillin/tazobactam or meropenem and clindamycin are currently recommended [11]. Investigation with a CT should be undertaken. Gas and fluid collection in soft tissue are suggestive of CM.

The importance of repeated surgical debridements cannot be overstated. Multiple incisions are often needed to achieve source control [12], [13]. If a limb is affected, fasciotomy of all compartments should be undertaken, not only for adequate debridement but also to counteract ischemia and subsequent risk for amputation. The wounds should not be closed to allow oxygenation to create a hostile environment for the anaerobe *C. septicum*. Some surgeons advocate packing the wounds with gauze soaked in diluted hydrogen peroxide to further increase the inhospitality.

HBOT may be a valuable adjunct in the treatment of CM. HBOT has been demonstrated to suppress the production of clostridial toxins, enhance the antibiotic effect, increase oxygen levels in tissue, and limit the extent of necrosis [14]. A systematic review of case studies indicated that using HBOT in the treatment of hypoxic wounds reduced mortality and amputation rates [15]. However, a Cochrane review from 2015 found insufficient evidence to support the use of HBOT in the treatment of gas gangrene. Therefore, the decision to use HBOT should be made based on experience and availability [9].

Caution must be taken when applying the discussed treatment principles to the elderly, as specific guidelines for this population are lacking.

## 4 Conclusion

CM has a high mortality rate if not promptly treated with antibiotics and surgery. This report describes a rare case of presumably injury-related *C. septicum* myonecrosis with a successful outcome.

## Consent

Written informed consent was obtained from the patient for publication of this case report and accompanying images. A copy of the written consent is available for review by the Editor-in-Chief of this journal on request.

## Ethical approval

Ethical approval is exempt/waived at our institution.

## Funding

No funding was received for this work.

## Guarantor

Måns Muhrbeck (MM).

## Research registration number

1. Name of the registry: Not applicable
2. Unique identifying number or registration ID: Not applicable
3. Hyperlink to your specific registration (must be publicly accessible and will be checked): Not applicable.

## CRediT authorship contribution statement

Salik Hamid (SH): Data collection, drafted and approved the final article.
Ashok Gadré (AG): Data collection, data analysis, revised and approved the final article.
Liselott Fornander (LF): Data analysis, revised and approved the final article.
Johanna Sjöwall (JS): Data analysis, revised and approved the final article.
Måns Muhrbeck (MM): Data collection and data analysis. Drafted, revised, and approved the final article.

## Declaration of competing interest

The authors have no conflicting interests to declare.
